# Deep Learning–Based Estimated Pulmonary Biological Age From Chest Computed Tomography Images in Healthy Adults: Model Development and Validation Study

**DOI:** 10.2196/78243

**Published:** 2026-03-12

**Authors:** Liping Zuo, Na Zhu, Bowen Wang, Donglai Li, Jinlei Fan, Zhaolei Fan, Yongsheng Shang, Yongxiang Wang, Lei Xu, Peng Zhou, Wangshu Cai, Dexin Yu

**Affiliations:** 1 Department of Radiology, Qilu Hospital of Shandong University, 107 Wenhua Xilu, Lixia District, Jinan 250012, Shandong, China, 86 18560081629; 2 Department of Clinical Laboratory, Qilu Hospital of Shandong University (Qingdao), Qingdao 266035, Shandong, China; 3 Department of Emergency Medicine, Qilu Hospital of Shandong University, Shandong Provincial Clinical Research Center for Emergency and Critical Care Medicine, Jinan 250012, Shandong, China; 4 Msun Health Technology Group Co., Ltd, Jinan 250014, Shandong, China; 5 Medical Imaging Department, Shengli Oilfield Central Hospital, Dongying 257100, Shandong, China; 6 Department of Radiology, Central Hospital Affiliated to Shandong First Medical University, Jinan 250013, Shandong, China

**Keywords:** estimated pulmonary biological age, chest computed tomography, chest CT, deep learning, age gap, chronic obstructive pulmonary disease

## Abstract

**Background:**

Estimated pulmonary biological age (ePBA) has emerged as a more reliable indicator for disease progression and mortality than chronological age, with chest computed tomography (CT) as a promising tool for calculating ePBA. However, the lack of models trained and validated with large-scale healthy adults hinders the generalizability of the CT-based ePBA.

**Objective:**

This study aims to develop an aging biomarker (ePBA) from multicenter chest CTs of healthy adults using deep learning and investigate the association between age gap (ePBA - chronological age) and pulmonary function as well as all-cause mortality in patients with chronic obstructive pulmonary disease (COPD).

**Methods:**

We used 11,187 chest CT scans from healthy adults at 3 health management centers and used multiple deep learning models. Of these, 7726 scans from institution A were used for model development. The remaining CT scans from institutions B (n=1506) and C (n=1955) served as external test datasets. To examine whether ePBA provided information beyond chronological age in patients with the disease, we investigated the association of age gap with lung function and all-cause mortality among 138 patients with COPD hospitalized at the same time period in institution A.

**Results:**

The deep learning models demonstrated acceptable applicability for this task and exhibited a strong correlation between ePBA and chronological age. Age gap was significantly associated with forced expiratory volume in 1 second expressed as percentage of predicted values reduction (*r_s_*=−0.18; *P*=.03) and an increased risk of all-cause mortality (hazard ratio: 1.16, 95% CI 1.08-1.25) in patients with COPD.

**Conclusions:**

This study developed and validated a biomarker of aging—ePBA—with deep learning models based on chest CT. Age gap could serve as a novel clinical biomarker in patients with COPD.

## Introduction

With the acceleration in global population aging, people who have chronic respiratory diseases have increased in number, and chronic respiratory disease has become the third leading cause of death, causing substantial economic and health burdens to society. Longitudinal studies have emphasized the critical need to precisely assess pulmonary senescence [1,2], which is essential for extending health span and reducing age-related respiratory morbidity. Thus, there is an urgent necessity to develop a biomarker of aging—estimated pulmonary biological age (ePBA)—which is applicable to large-scale populations.

Models have been developed to estimate ePBA based on genes, proteins, and cells, but their expensive and complex assays prevent clinical translation [3-6]. Imaging techniques, such as noninvasive and highly accessible diagnostic tools, are widely used across health care settings, from primary care to tertiary medical centers. Despite the common use of chest radiographs for initial respiratory assessments, their limited spatial resolution contrasts with the detailed structural and physiological insights provided by computed tomography (CT) scans. Chest CT offers a detailed assessment of changes in lung parenchyma, small airways, and pulmonary vasculature associated with declining lung function and aging [7,8]. Therefore, an ePBA assessment model based on chest CT is both highly feasible and generalizable, holding great promise for managing chronic respiratory diseases [6].

Deep learning can automatically extract features from massive training data, which is helpful for exploring the signs of aging in medical images. Although Azarfar et al [9] have explored a deep learning model for estimating biological age using chest CT, it was trained on the National Lung Screening Trial dataset, with participants having a smoking history of at least 30 pack-years and comorbidities and thus could not represent a healthy elderly population. In a previous study [10], ePBA was calculated using chest CT reconstructed bone from 2500 healthy participants, but due to the single-center nature and limited sample size in the validation dataset, the stability and generalization of the model needed to be improved. Thus, the lack of models trained and validated with large-scale healthy adults hinders the generalizability of the CT-based ePBA.

This study aimed to develop and validate ePBA using deep learning models based on chest CT scans from healthy individuals. The age gap—defined as the difference between ePBA and chronological age—was used to represent the extent of lung aging in an individual relative to a standard population. Furthermore, we examined the correlation between age gap and pulmonary function in patients with chronic obstructive pulmonary disease (COPD) and evaluated its ability to assess all-cause mortality risk in these patients, which may have potential applications for the management of patients with chronic respiratory diseases.

## Methods

### Participants in Model Development and Test

Participants underwent chest CT scans between January 2022 and November 2023 as periodic health check-ups in 3 health management centers (Qilu Hospital of Shandong University, Jinan [institution A]; Central Hospital Affiliated to Shandong First Medical University, Jinan [institution B]; and Shengli Oilfield Central Hospital, Dongying [institution C]), who were enrolled to develop and validate a deep learning model for calculating ePBA.

**Figure 1.**
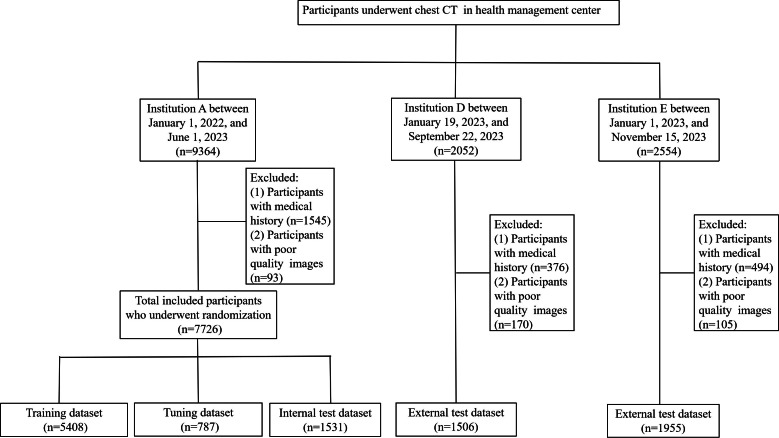
The flowchart of the included and excluded individuals in model development and test. CT: computed tomography.

If participants underwent multiple chest CT scans during the data collection period, only the initial scan was selected. Disease registration was completed for all participants on the day of the CT examination at the 3 hospitals. The disease registration was established in accordance with the International Classification of Diseases and Related Health Problems, Tenth Revision [11,12]. Participants with disease registrations or compromised poor image quality were excluded (Figure 1).

### Ethical Consideration

This retrospective and multicenter study was approved by the ethics committee of Qilu Hospital of Shandong University. No invasive operations were used or human tissue was obtained in this study, so the requirement for informed consent was waived (KYLL-202310‐027). The study was conducted in accordance with the principles of the Declaration of Helsinki. All chest CT images and clinical data were deidentified and anonymized prior to analysis to protect patient privacy. The confidentiality of participants’ personal information was strictly maintained throughout the study. As this was a retrospective study, participants were not compensated.

### CT Acquisition

Noncontrast CT images of healthy participants were acquired following complete inspiration. Images of the entire lung, with a slice thickness of 1‐5 mm, were reconstructed using standard kernels. The CT scanners used were Siemens Force, SOMATOM Definition AS (both from Siemens Healthineers), Philips Incisive CT, Philips Ingenuity CT, Philips IQon Spectral CT (all from Philips Healthcare), United Imaging uCT710 (United Imaging Healthcare), GE Revolution CT, and GE Discovery CT750 HD (both from GE Healthcare). The scanning parameters are shown in Table S1 in Multimedia Appendix 1.

### Ground Truth Labeling and Data Partition

Chest CT images of healthy participants were labeled using chronological age. All labeled CTs from institution A were randomly assigned in a 7:1:2 ratio into training, tuning, and internal test datasets, and age distribution consistency was preserved. The tuning dataset was dedicated to hyperparameter optimization, whereas the internal test dataset evaluated preliminary model performance. Additionally, the labeled CTs from institutions B and C were used for external test datasets.

### Model Development and Test

**Figure 2.**
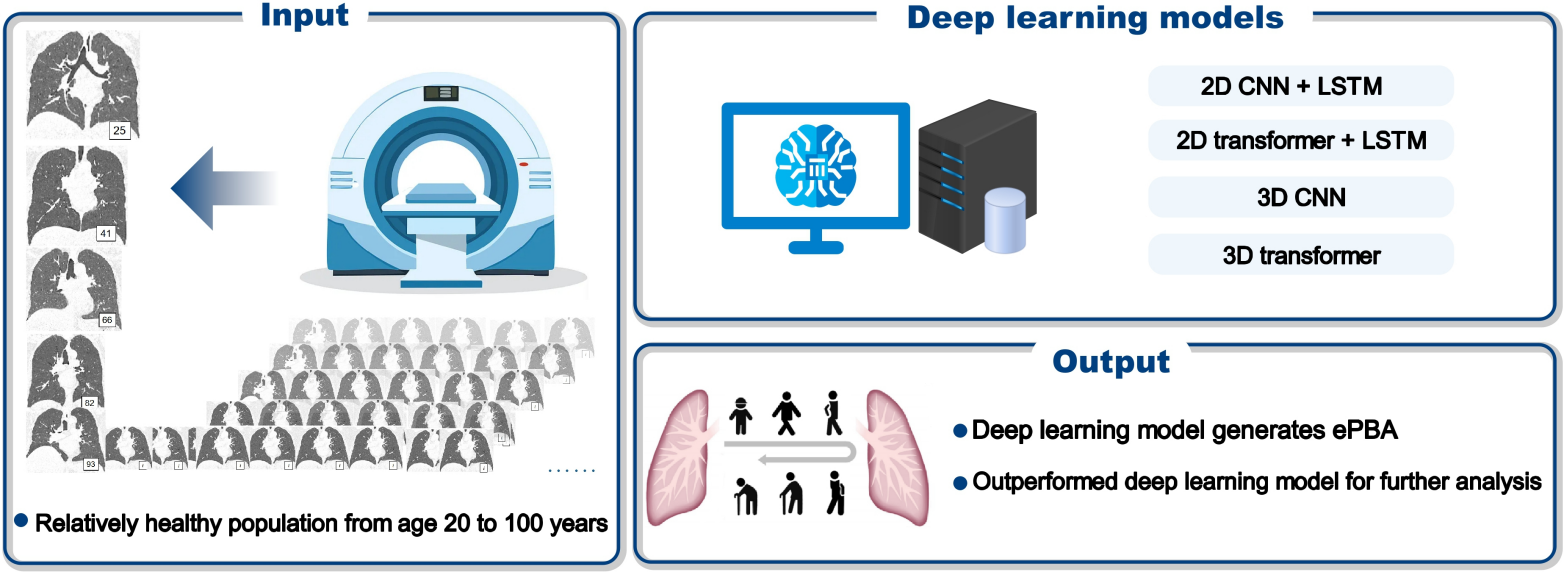
Development of deep learning estimated pulmonary biological age (ePBA) models. Deep learning–based ePBA models were developed using chest computed tomography (CT) scans from relatively healthy individuals aged 20 to 100 years with chronological age labels. CNN: convolutional neural network; LSTM: long short-term memory.

A total of 10 deep learning models (visual geometry group [11 layers] [VGG11] [13]+long short-term memory [LSTM]; residual network (18 layers) [14]+LSTM; ConvNeXt [15]+LSTM; vision transformer [16]+LSTM; shifted windows transformer [17]+LSTM; 3D VGG11 [18]; 3D residual network (18 layers) [19]; 3D ConvNeXt [15]; 3D vision transformer [20]; 3D shifted windows transformer [21]), including 4 major categories (2D convolutional neural network [CNN]/transformer+LSTM, 3D CNN or transformer), were trained to calculate ePBA from healthy individuals (Figure 2). Further details on model training and parameter settings are available in supplementary methods in Multimedia Appendix 1. The model most suitable for this task was selected for subsequent analysis.

The training and tuning datasets were used to train and tune this model. Data augmentation included random horizontal flips, vertical flips, rotations, and offsets to increase the diversity of the data. The loss function used mean square error, and the optimizer used Adam. The initial learning rate was set to 10⁻⁴. We used a cosine annealing schedule over 500 epochs and used early stopping to prevent overfitting. Through training and tuning, we obtained an optimal model. The performance of the optimal model was evaluated on internal and external test datasets. To empirically validate the computational efficiency and performance of the 2D CNN+LSTM framework relative to 3D CNNs, we extended our ablation studies to a suite of 3D convolutional architectures. The input processing for these 3D models aligns with that of the 2D pipeline, ensuring fair comparisons across architectures.

The development process was performed using the PyTorch framework (version 1.8.1), and the hardware was an NVIDIA TITAN V GPU with 12 GB of memory.

To investigate the decision-making process of our best-performing model, we used score-weighted class activation mapping (score-CAM) [22] to visualize the discriminative regions it focused on during validation. This method represents a novel gradient-free visual interpretation technique aimed at explaining the classification decisions made by deep learning models. By highlighting important image regions in the visualizations, score-CAM reveals where the model allocates attention during the classification process. Notably, it serves as a bridge between perturbation-based and CAM-based approaches while providing intuitive and accessible insights into how the activation map weights are derived.

### Application of ePBA in Patients with COPD

To evaluate the clinical applicability of the ePBA, we retrospectively enrolled patients hospitalized with COPD at institution A between January 1, 2022 and June 1, 2023. Data on demographics, pulmonary function, and medical history were collected from medical records. Dyspnea severity was assessed using the modified Medical Research Council (mMRC) scale. We also evaluated the ADO (age, dyspnea, and airflow obstruction) index [23], an abbreviated version of the BODE index (including body-mass index, airflow obstruction, dyspnea, and exercise capacity) and a simpler COPD prognostic risk index based on age, dyspnea, and airflow obstruction. Chest CT images from patients with COPD were input into the ePBA model, and the age gap (the difference between ePBA and chronological age) was subsequently calculated. Age gap above 0 indicated a higher level of aging than their healthy peers of the same chronological age, while age gap below 0 indicated a lower level of aging. The vital status was collected by reviewing medical records or through telephone calls to the patient or family. After at least 6 months of follow-up, a total of 138 patients with COPD were included in the study. The detailed inclusion and exclusion criteria are described in Figure S1 in Multimedia Appendix 1. An investigation was conducted to determine whether the age gap was related to the results of pulmonary function measurements and their risk of death.

### Statistical Analysis

Categorical variables were presented as numbers and percentages. Continuous variables were summarized as mean (SD) or median and IQR, based on their distribution. Group comparisons were performed using the 2-tailed *t* test or Mann-Whitney *U* test for continuous variables and the chi-square or Fisher exact test for categorical variables, as appropriate.

The Pearson correlation coefficient (*r*), coefficient of determination (*R*^2^), mean absolute error (MAE), mean square error, and root mean square error between the ePBA and chronological age were calculated to evaluate the regression performance of the deep learning model. The 99% CIs for these metrics were estimated using the nonparametric bootstrap method with 1000 replicates. This process involved generating 1000 independent bootstrap samples through random resampling with replacement from the original dataset, each of the same size as the original. The CIs were then derived using the percentile method, based on the 0.5th and 99.5th percentiles of the bootstrap sampling distribution.

In patients with COPD, the correlation between age gap and pulmonary function measurements was calculated. To examine the relationship between age gap and the mortality risk in patients with COPD, Cox proportional hazards regression models were conducted with age gap used as a continuous variable and a binary variable, respectively (biologically older and younger represent age gap >0 and ⩽0 [24], respectively). Adjustments were made for chronological age, sex, mMRC scale, smoking status, and medical history. Variance inflation factor (VIF) testing was performed to assess multicollinearity among the included variables. The proportional hazard assumption for the Cox model was checked using Schoenfeld residuals. For survival analysis, to assess the discriminatory ability of the age gap and ADO index, we performed Harrell concordance index and area under the curve, with 95% CIs yielded by 1000 bootstrap resamples. To visualize the discriminative performance of age gap, we also plotted the Kaplan-Meier curves of the biologically older and younger groups.

Statistical analyses were performed using the R software (version 3.3.3; R Foundation for Statistical Computing) and SPSS 26.0 for Windows (IBM Corp.). Statistical significance was set at *P*<.05.

## Results

### Demographic Characteristics of Healthy Individuals

A total of 13,970 participants with health check-ups (9364 in institution A, 2052 in institution B, and 2554 in institution C) were eligible for inclusion; 11,187 (7726 in institution A, 1506 in institution B, and 1955 in institution C) were ultimately enrolled after exclusion (Figure 1). Participants from institution A were randomly assigned to the training (n=5408, 2588 male participants), tuning (n=787, 385 male participants), and internal test datasets (n=1531, 710 male participants) in a ratio of 7:1:2. Additionally, participants from institutions B (n=1506, 814 male participants) and C (n=1955, 1310 male participants) were assigned to the external test datasets. The median ages (quartiles) of male and female participants were similar in the training, tuning, internal validation test, and the external test datasets (Table 1).

**Table 1.** Demographic characteristics of individuals.

Characteristics	Training dataset	Tuning dataset	Internal test dataset	External test dataset	
	Institution A	Institution A	Institution A	Institution B	Institution C
Total individuals	5408	787	1531	1506	1955
Male, n (%)	2588 (47.9)	385 (48.9)	710 (46.4)	814 (54.1)	1310 (67)
Female, n (%)	2820 (52.1)	402 (51.1)	821 (53.6)	692 (45.9)	645 (33)
Age (y), (min–max)					
Male	53 (20-98)	53 (22-100)	54 (23-94)	54 (20-97)	47 (21-85)
Female	53 (20-97)	53 (22-98)	54 (22-96)	54 (21-94)	47 (23-83)

### Performance of Deep Learning Models for Age Estimation

**Figure 3.**
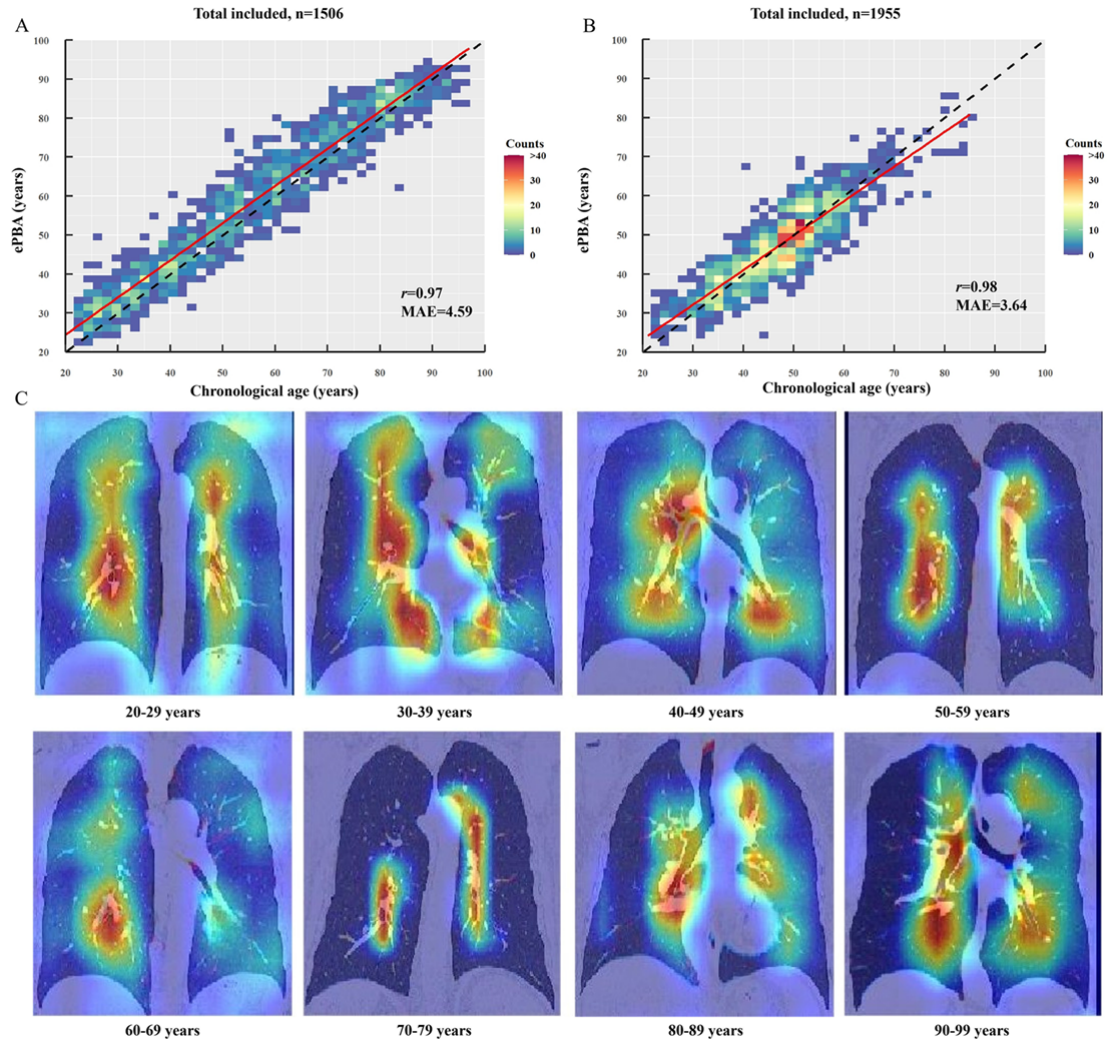
Scatterplot between estimated pulmonary biological age (ePBA) derived from the VGG11-LSTM deep learning model and chronological age in (A) institution B and (B) institution C. (C) Score-weighted class activation mapping (score-CAM) original image class activation maps for healthy individuals. LSTM: long short-term memory; MAE: mean absolute error; VGG11: visual geometry group (11 layers).

All deep learning models achieved acceptable performance in this task (Table 2, Table S2-S11 in Multimedia Appendix 1). In total healthy individuals, the ePBAs derived from the VGG11+LSTM deep learning model were significantly associated with chronological ages across all datasets (training, tuning, internal test, and external test; all *r* >0.95, Table S2 in Multimedia Appendix 1). The external test dataset showed a strong correlation between ePBA and chronological age (*r*=0.97, 99% CI 0.96-0.97 in institution B, Figure 3A; *r*=0.98, 99% CI 0.96-0.99 in institution C, Figure 3B) and good model performance (institution B: *R*^2^=0.93, 99% CI 0.92-0.94, MAE=4.59, 99% CI 4.34-4.83; institution C: *R*^2^=0.93, 99% CI 0.84-0.94, MAE=3.64, 99% CI 3.47-3.81). Notably, the VGG11-LSTM model also exhibited a favorable balance between complexity and inference efficiency, containing 134.02 million parameters and achieving an average inference time of 18 ms per sample, rendering it suitable for potential clinical deployment. Thus, this model most suitable for this task was selected for subsequent analysis in patients with COPD.

**Table 2.** Regression performance parameters in the external test dataset, parameter counts, and inference time of deep learning models^a^.

Model and backbone		*r*		*R* ^2^		MAE^b^		MSE^c^		RMSE^d^		Parameters (million)	Inference (ms)
		Institution B	Institution C	Institution B	Institution C	Institution B	Institution C	Institution B	Institution C	Institution B	Institution C		
2D CNN+LSTM^f^^g^													
VGG11^e^		0.97 (0.96-0.97)	0.98 (0.96-0.99)	0.93 (0.92-0.94)	0.93 (0.84-0.94)	4.59 (4.34-4.83)	3.64 (3.47-3.81)	34.46 (34.12-34.80)	21.64 (21.37-21.91)	5.87 (5.53-6.21)	4.65 (4.38-4.92)	134.02	18
Resnet18^h^		0.97 (0.96-0.97)	0.88 (0.87-0.89)	0.93 (0.92-0.94)	0.78 (0.75-0.80)	5.00 (4.76-5.24)	3.80 (3.63-3.97)	38.01 (37.66-38.36)	22.77 (22.52-23.03)	6.17 (5.82-6.51)	4.77 (4.52-5.03)	14.59	6
ConvNeXt		0.95 (0.95-0.96)	0.83 (0.81-0.85)	0.91 (0.90-0.92)	0.69 (0.66-0.72)	6.78 (6.46-7.10)	4.86 (4.65-5.08)	68.96 (68.57-69.36)	37.48 (37.15-37.80)	8.30 (7.91-8.70)	6.12 (5.80-6.45)	31.37	24
2D transformer+LSTM													
ViT^i^		0.95 (0.95-0.96)	0.85 (0.84-0.87)	0.90 (0.89-0.91)	0.66 (0.62-0.69)	5.02 (4.80-5.27)	4.32 (4.12-4.52)	39.53 (36.30-43.38)	29.59 (26.75-32.26)	6.29 (6.03-6.21)	5.44 (5.17-5.68)	25.02	19
Swin transformer		0.96 (0.96-0.97)	0.88 (0.86-0.89)	0.89 (0.88-0.91)	0.73 (0.69-0.76)	5.07 (4.83-5.36)	3.78 (3.61-3.95)	41.05 (37.02-45.95)	23.37 (21.50-25.56)	6.41 (6.08-6.78)	4.83 (4.64-5.06)	31.07	32
3D CNN													
VGG11		0.95 (0.95- 0.96)	0.85 (0.83- 0.86)	0.91 (0.89- 0.92)	0.58 (0.51- 0.63)	4.58 (4.32- 4.85)	4.77 (4.56- 4.99)	35.34 (31.65, 39.98)	36.37 (33.12- 40.38)	5.95 (5.63- 6.32)	6.03 (5.75- 6.35)	178.66	20
Resnet18		0.95 (0.94- 0.96)	0.84 (0.83- 0.86)	0.90 (0.89- 0.91)	0.61 (0.55- 0.66)	4.90 (4.65- 5.17)	4.56 (4.35- 4.77)	38.54 (34.95- 42.74)	33.81 (30.74- 37.25)	6.21 (5.91- 6.54)	5.82 (5.54-6.10)	33.21	64
ConvNeXt		0.89 (0.88- 0.91)	0.71 (0.67- 0.74)	0.80 (0.77- 0.82)	0.10 (0.04- 0.22)	6.80 (6.46- 7.19)	6.78 (6.47- 7.12)	78.01 (68.96- 87.46)	77.56 (70.11- 85.93)	8.83 (8.30- 9.35)	8.81 (8.37- 9.27)	29.78	197
3D transformer													
ViT		0.86 (0.85- 0.88)	0.64 (0.60- 0.67)	0.74 (0.71- 0.77)	0.01 (0.14- 0.12)	8.04 (7.62- 8.44)	7.33 (7.02- 7.67)	99.82 (90.17- 110.19)	86.75 (79.62- 94.39)	9.99 (9.50- 10.50)	9.31 (8.92- 9.72)	53.85	9
Swin transformer		0.92 (0.91- 0.93)	0.78 (0.75- 0.80)	0.85 (0.83- 0.87)	0.34 (0.23- 0.42)	5.87 (5.55, 6.20)	5.90 (5.63- 6.17)	57.80 (51.21- 64.28)	57.21 (52.05- 63.42)	7.60 (7.16- 8.02)	7.56 (7.21- 7.96)	9.72	15

a All the data in parentheses are 99% CI.

b MAE: mean absolute error.

c MSE: mean square error.

d RMSE: root mean square error.

e VGG11: visual geometry group (11 layers).

f CNN: convolutional neural network.

g LSTM: long short-term memory.

h ResNet18: residual network (18 layers).

i ViT: vision transformer.

To identify critical regions for optimizing feature localization during model training, we performed a visualization analysis using score-CAM. Figure 3C shows the score-CAM original image class activation maps of ePBA derived from VGG11-LSTM. The highlighted important image regions were located around the tracheal and bronchial vascular bundles after the visualization in healthy individuals at different age group distribution.

### Application of ePBA in Patients With COPD

**Figure 4.**
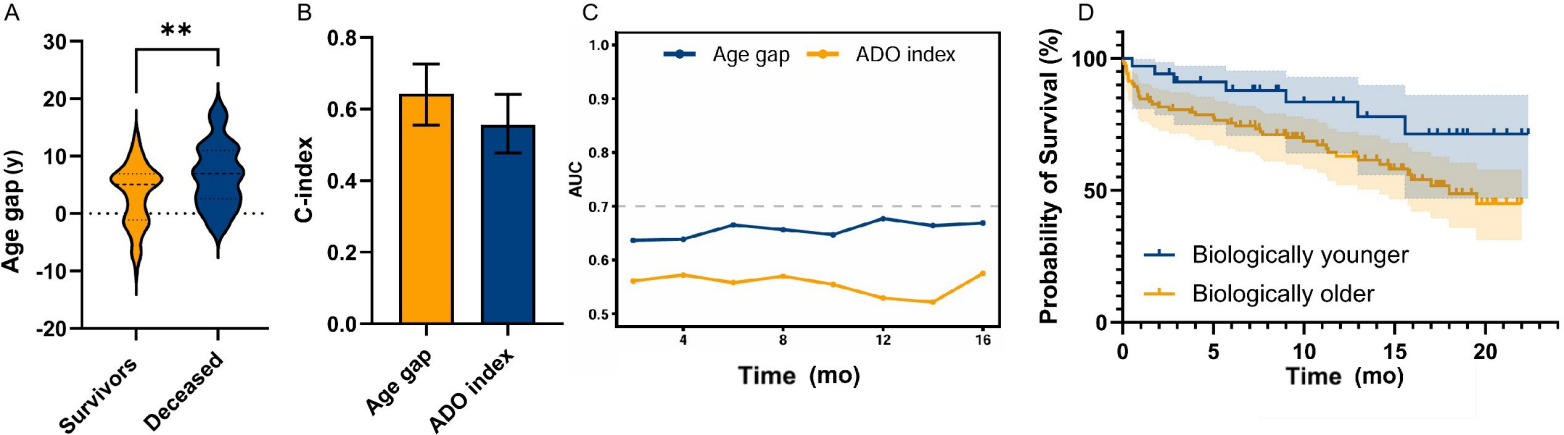
(A) The distribution of age gap between survivors and deceased patients with chronic obstructive pulmonary disease (COPD); (B) comparison of the performance of age gap and ADO (age, dyspnea, and airflow obstruction) index in predicting the survival outcome of patients with COPD; (C) time-dependent area under the curve (AUC) curves for age gap and ADO index in patients with COPD; and (D) Kaplan-Meier curve of overall survival for biologically younger and older groups in patients with COPD. **P<.01.

The ePBA of the 138 patients with COPD was significantly greater than their chronological age (ePBA: median 78.65, IQR 74.11-84.00 y vs chronological age: median 74.00, IQR 68.00-81.00 y; *P*<.001), resulting in a median age gap of median 5.43 (IQR 0.49-7.79) years, which was also significantly greater than 0 (*P*<.001). These findings indicate that these patients are in a state of accelerated aging. Table 3 provides the demographics, pulmonary function measurements, and medical history of survivors and deceased patients with COPD. As shown in Figure 4A, the age gap of deceased patients with COPD (median 6.96, IQR 3.00-11.00 y) was significantly greater than that of survivors (median 5.05, IQR –1.04 to 6.89 y; *P*=.004), and the mMRC scale score (median 4.00, IQR 3.25-4.00) was also significantly higher than that of survivors (median 3.00, IQR 2.00-4.00; *P*<.001; Table 3). There were no significant differences in other aspects.

**Table 3.** Demographics, pulmonary function, ADO (age, dyspnea, and airflow obstruction) index, and medical history in survivors and deceased patients with chronic obstructive pulmonary disease (COPD).

Characteristics	All (n=138)	Survivors (n=88)	Deceased (n=50)	Statistics	*P* value
Chronological age (y), median (Q1, Q3)	74.00 (68.00 to 81.00)	74.00 (69.00 to 81.00)	73.50 (66.00 to 82.00)	–0.55^a^	.58
ePBA^b^ (y), median (Q1, Q3)	78.65 (74.11 to 84.00)	77.66 (73.09 to 83.11)	79.97 (76.23 to 85.00)	–1.79^a^	.07
Age gap (y), median (Q1, Q3)	5.43 (0.49 to 7.79)	5.05 (–1.04 to 6.89)	6.96 (3.00 to 11.00)	–2.85^a^	.004
Male participants, n (%)	115 (83.3)	74 (84.1)	41 (82.0)	0.10 (*df*=1)^c^	.75
BMI (kg/m^2^), median (Q1, Q3)	22.47 (20.25 to 24.74)	22.13 (20.53 to 24.38)	22.96 (19.42 to 25.06)	–0.58^a^	.56
Smoking status, n (%)				3.80 (*df*=2)^c^	.15
Never smoker	37 (26.8)	25 (28.4)	12 (24.0)		
Current smoker	41 (29.7)	30 (34.1)	11 (22.0)		
Ex-smoker	60 (43.5)	33 (37.5)	27 (54.0)		
mMRC^d^, median (Q1, Q3)	4.00 (3.00 to 4.00)	3.00 (2.00 to 4.00)	4.00 (3.25 to 4.00)	3.77^a^	<.001
FEV1%^e^, median (Q1, Q3)	0.42 (0.33 to 0.55)	0.44 (0.34 to 0.56)	0.38 (0.31 to 0.54)	–1.52^a^	.13
FVC%^f^, median (Q1, Q3)	0.67 (0.51 to 0.79)	0.70 (0.50 to 0.80)	0.65 (0.51 to 0.74)	–0.91^a^	.36
FEV1 or FVC^h^^, g^ , median (Q1, Q3)	0.50 (0.43 to 0.59)	0.51 (0.46 to 0.60)	0.49 (0.42 to 0.58)	–1.48^a^	.14
ADO index, median (Q1, Q3)	7.00 (5.00 to 8.00)	6.50 (5.00 to 8.00)	7.00 (6.00 to 8.00)	–2.11^a^	.03
Medical history, n (%)					
Hypertension	68 (49.3)	42 (47.7)	26 (52.0)	0.23 (*df*=1)^c^	.63
Diabetes	22 (15.9)	14 (15.9)	8 (16.0)	0.00 (*df*=1)^c^	.99
Cerebrovascular disease	25 (18.1)	18 (20.5)	7 (14.0)	0.90 (*df*=1)^c^	.34
Follow-up period (mo), median (Q1, Q3)	9.97 (4.83 to 16.93)	13.40 (8.68 to 18.42)	4.50 (0.76 to 11.26)	–5.78^a^	<.001

a *z*-score.

b ePBA: estimated pulmonary biological age.

c Chi-square.

d mMRC: modified Medical Research Council scale.

e FEV1%: forced expiratory volume in 1 second expressed as percentage of predicted values.

f FVC%: forced vital capacity expressed as percentage of predicted values.

g FEV1: forced expiratory volume in 1 second.

h FVC: forced vital capacity.

Spearman correlation analysis showed that age gap was negatively correlated with forced expiratory volume in 1 second expressed as percentage of predicted values (FEV1%; *r_s_*=−0.18; *P*=.03), while no significant correlation was observed between age gap and forced vital capacity expressed as percentage of predicted values (*r_s_*=−0.11; *P*=.21) or FEV1 or forced vital capacity (*r_s_*=−0.14; *P*=.09).

Before the multivariate Cox regression analysis, VIF testing was performed to assess multicollinearity among the included variables (Table S12 in Multimedia Appendix 1). As VIF values above 10 suggest significant multicollinearity, all variables demonstrated VIF values below 2, indicating the absence of severe multicollinearity. These results confirm the reliability and validity of the subsequent multivariate regression models for evaluating the independent association between age gap and mortality in patients with COPD. In the Cox regression analysis model, age gap was significantly associated with an increased risk of mortality in patients with COPD (hazard ratio [HR]: 1.12, 95% CI 1.06-1.19). Further adjustments for chronological age, sex, mMRC scale, smoking status, and medical history showed similar HR (HR: 1.16, 95% CI 1.08-1.25; Table 4). Compared with biologically younger patients, biologically older patients had a significantly higher risk of death (HR: 2.24, 95% CI 1.01-4.98), and this remained significant even after adjusting for covariates (HR: 2.78, 95% CI 1.17-6.58; Table 4). The Schoenfeld residuals test results (Table S13 in Multimedia Appendix 1) indicated that both the global test and individual covariates in the Cox proportional hazards models of this study satisfied the proportional hazards assumption (*P*>.05). The global Schoenfeld residuals test *P* values for model 1, model 2, and model 3 were .39, .75, and .42, respectively. The concordance index of age gap was 0.643 (95% CI 0.555-0.726), which was higher than the ADO index 0.556 (95% CI 0.478-0.641) in differentiating the overall survival rate of patients with COPD (Figure 4B). Age gap maintained a consistently high area under the curve value throughout the entire prediction interval (Figure 4C). The Kaplan-Meier overall survival curve demonstrated an excellent distinction in the risk of death between the younger and older biological age groups, and the survival time of the older biological age group was significantly shorter than that of the younger biological age group (*P*=.047; Figure 4D).

**Table 4.** Association between age gap and mortality risk in patients with chronic obstructive pulmonary disease (COPD)^a^.

	Model 1^b^	*P* value	Model 2^c^	*P* value	Model 13^d^	*P* value
Age gap (y)	1.12 (1.06-1.19)	<.001	1.16 (1.08-1.25)	<.001	1.16 (1.08-1.25)	<.001
Age gap category						
Biologically younger	Reference		Reference		Reference	
Biologically older	2.24 (1.01-4.98)	.048	2.72 (1.16-6.35)	.02	2.78 (1.17-6.58)	.02

a Biologically older and younger represent age gap >0 and ≤0, respectively. All data in parentheses are 95% CI.

b Unadjusted.

c Adjusting for chronological age, sex, BMI, and the modified Medical Research Council scale.

d Adjusting for covariates in model 2 plus smoking status, and medical history (hypertension, diabetes, and cerebrovascular disease).

## Discussion

In this study, we developed and validated multiple deep learning models to calculate ePBA using chest CT scans from healthy individuals. Second, when applied to a COPD cohort, the derived “age gap” (ePBA—chronological age) emerged as a significant clinical biomarker, showing a negative correlation with FEV1% and proving to be a stronger predictor of all-cause mortality than the established ADO index.

In previous studies on calculating ePBA, gene-based, protein-based, and cellular marker–based ePBA calculation models have been reported [24-26]. However, due to the high cost and complex workflow, these models were not suitable for large-scale cohort applications and were more suitable for exploring the biological mechanisms of lung aging. Liang et al [4] developed an estimation equation for lung age based on pulmonary function indicators (FEV1, FEF50%, FEF75%, and height) by the spline method (male: *R*^2^=0.661, female: *R*^2^=0.690). This equation might have a higher estimation error, as the coefficient of determination of this equation was lower than that of the CT-based deep learning model in this study. Furthermore, they failed to accomplish widespread clinical application due to the great difficulty in conducting pulmonary function tests in primary medical units. Among imaging tests, X-ray is often used as the first-line recommended test for chest disease. Mitsuyama et al [6] developed a biomarker of aging from chest radiography and examined the correlation between estimated age and chronological age (*r*=0.95). However, due to the low-density resolution of chest X-ray, chest CT is preferred for the full characterization of the aging lung in identifying signs of lung aging. The chest imaging features related to aging show vascular curvature and calcification, mediastinal lipomatosis, diaphragmatic bulge and protrusion, and musculoskeletal features, such as chest osteophyte and costal cartilage calcification [27]. Azarfar et al [9] developed an age estimation model using chest CT and deep learning and demonstrated its association with the risk of lung cancer. However, this model was trained using the National Lung Screening Trial dataset, which contains a large number of heavy smokers, and the participants have comorbidities, such as chronic lung diseases, making it difficult for this model to be generalized to patients with chronic respiratory diseases. In the study of Lu et al [10], ePBA was calculated using chest CT reconstructed bone from 2500 healthy participants, but due to the single-center nature and limited sample size in the validation dataset (n=200), the stability and generalization of the model needed to be improved. In this study, chest CT images of 11,187 healthy adults from 3 institutions were used to train the model to ensure that the model training dataset could represent a healthy population, and it was externally validated on 3461 healthy adults from 2 institutions that were included in the external test datasets, ensuring its stability and generalizability.

Importantly, we verified the application of ePBA in patients with COPD. It was observed that an increase in the ePBA-derived age gap was significantly associated with a reduction in FEV₁% in patients with COPD. A similar negative association with lung function was also observed for phenotypic age acceleration, which was derived from phenotypic age based on chronological age and 9 clinical biomarkers as described by Wang et al [24]. After adjusting for chronological age and other covariates, the age gap remained independently associated with an elevated risk of all-cause mortality in patients with COPD. Notably, the predictive performance of age gap for mortality surpassed that of the established ADO index. Given that ePBA can be derived solely from routinely acquired chest CT images without requiring additional clinical parameters, it shows promise for improving the management of patients with chronic respiratory diseases and the implementation of targeted interventions aimed at addressing aging-related issues.

There are several limitations in this study. First, we did not compare this model with some established biological age markers (eg, epigenetic clock, DNAmGrimAge) due to the complexity and higher costs. Despite the continued refinement of epigenetic clocks over the last decade, standardized measures of organ age remain to be developed [28,29]. Second, participants were enrolled in China from a single ethnic background. The inclusion of participants with diverse ethnic, demographic, and socioeconomic backgrounds limits the generalizability of our current findings. Third, we did not construct a hybrid deep learning model based on clinical phenotype and chest CT. This was because clinical data, such as pulmonary function tests, are typically not collected during routine health check-ups, limiting their availability for model development. Finally, the COPD validation cohort has certain limitations, including its relatively small size, short follow-up, and predominance of severe cases. Although suitable for assessing mortality risk, this composition limits the direct evaluation of ePBA’s ability to distinguish early pathological pulmonary aging from healthy aging. Therefore, future studies in community-based cohorts with milder disease and longer follow-up are warranted to verify the model’s broader applicability.

In summary, this study constructed deep learning models to calculate the biomarker of aging—ePBA—based on chest CT images of multicenter healthy adults. The results showed that the age gap (ePBA—chronological age), representing the extent of lung aging in an individual relative to a standard population, may have potential applications for the management of patients with COPD.

## Supplementary material

10.2196/78243 Multimedia Appendix 1 Supplementary methods, results, and figures.

## Abbreviations

ADO: age, dyspnea, and airflow obstruction
CNN: convolutional neural network
COPD: chronic obstructive pulmonary disease
ePBA: estimated pulmonary biological age
FEV1: forced expiratory volume in 1 second
FEV1%: forced expiratory volume in 1 second expressed as percentage of predicted values
HR: hazard ratio
LSTM: long short-term memory
MAE: mean absolute error
mMRC: the modified Medical Research Council
Score-CAM: score-weighted class activation mapping
VGG11: visual geometry group (11 layers)
VIF: variance inflation factor

## References

[1] Schuliga M, Pechkovsky DV, Read J (2018). Mitochondrial dysfunction contributes to the senescent phenotype of IPF lung fibroblasts. J Cell Mol Med.

[2] Schafer MJ, Zhang X, Kumar A (2020). The senescence-associated secretome as an indicator of age and medical risk. JCI Insight.

[3] Bao H, Cao J, Aging Biomarker Consortium (2023). Biomarkers of aging. Sci China Life Sci.

[4] Liang X, Xie Y, Gao Y (2022). Estimation of lung age via a spline method and its application in chronic respiratory diseases. NPJ Prim Care Respir Med.

[5] Tian YE, Cropley V, Maier AB, Lautenschlager NT, Breakspear M, Zalesky A (2023). Heterogeneous aging across multiple organ systems and prediction of chronic disease and mortality. Nat Med.

[6] Mitsuyama Y, Matsumoto T, Tatekawa H (2023). Chest radiography as a biomarker of ageing: artificial intelligence-based, multi-institutional model development and validation in Japan. Lancet Healthy Longev.

[7] Nam JG, Kang HR, Lee SM (2022). Deep learning prediction of survival in patients with chronic obstructive pulmonary disease using chest radiographs. Radiology.

[8] Olesen ASO, Miger K, Ørting SN (2025). AI-based algorithm to detect heart and lung disease from acute chest computed tomography scans: protocol for an algorithm development and validation study. JMIR Res Protoc.

[9] Azarfar G, Ko SB, Adams SJ, Babyn PS (2024). Deep learning-based age estimation from chest CT scans. Int J Comput Assist Radiol Surg.

[10] Lu T, Diao YR, Tang XE (2023). Deep learning enables automatic adult age estimation based on CT reconstruction images of the costal cartilage. Eur Radiol.

[11] Weiner MG (2018). POINT: is ICD-10 diagnosis coding important in the era of big data? Yes. Chest.

[12] (2015). ICD-10: there’s a code for that. Lancet.

[13] Simonyan K, Zisserman A (2015). Very deep convolutional networks for large-scale image recognition. https://www.scirp.org/reference/referencespapers?referenceid=3764205.

[14] He K, Zhang X, Ren S, Sun J Deep residual learning for image recognition. https://ieeexplore.ieee.org/document/7780459.

[15] Liu Z, Mao H, Wu CY, Feichtenhofer C, Darrell T, Xie S A ConvNet for the 2020s. https://ieeexplore.ieee.org/document/9879745.

[16] Dosovitskiy A, Beyer L, Kolesnikov A, Weissenborn D, Houlsby N (2020). An image is worth 16x16 words: transformers for image recognition at scale. arXiv.

[17] Liu Z, Lin Y, Cao Y Swin transformer: hierarchical vision transformer using shifted windows. https://ieeexplore.ieee.org/document/9710580.

[18] Tran D, Bourdev L, Fergus R, Torresani L, Paluri M Learning spatiotemporal features with 3D convolutional networks. https://ieeexplore.ieee.org/document/7410867.

[19] Hara K, Kataoka H, Satoh Y (2018). Can spatiotemporal 3D CNNs retrace the history of 2D CNNs and ImageNet?. https://ieeexplore.ieee.org/document/8578783.

[20] Perera S, Navard P, Yilmaz A (2024). SegFormer3D: an efficient transformer for 3D medical image segmentation. https://www.computer.org/csdl/proceedings-article/cvprw/2024/654700e981/20A1jcB3z20.

[21] Cai Y, Long Y, Han Z (2023). Swin Unet3D: a three-dimensional medical image segmentation network combining vision transformer and convolution. BMC Med Inform Decis Mak.

[22] Li J, Zhang D, Meng B, Li Y, Luo L (2023). FIMF score‐CAM: fast score‐CAM based on local multi‐feature integration for visual interpretation of CNNs. IET Image Process.

[23] Puhan MA, Garcia-Aymerich J, Frey M (2009). Expansion of the prognostic assessment of patients with chronic obstructive pulmonary disease: the updated BODE index and the ADO index. Lancet.

[24] Wang T, Duan W, Jia X (2024). Associations of combined phenotypic ageing and genetic risk with incidence of chronic respiratory diseases in the UK Biobank: a prospective cohort study. Eur Respir J.

[25] Sanders JL, Putman RK, Dupuis J (2021). The association of aging biomarkers, interstitial lung abnormalities, and mortality. Am J Respir Crit Care Med.

[26] Hernandez Cordero AI, Yang CX, Milne S (2021). Epigenetic blood biomarkers of ageing and mortality in COPD. Eur Respir J.

[27] Ensor RE, Fleg JL, Kim YC, de Leon EF, Goldman SM (1983). Longitudinal chest X-ray changes in normal men. J Gerontol.

[28] Elliott ML, Caspi A, Houts RM (2021). Disparities in the pace of biological aging among midlife adults of the same chronological age have implications for future frailty risk and policy. Nat Aging.

[29] McGreevy KM, Radak Z, Torma F (2023). DNAmFitAge: biological age indicator incorporating physical fitness. Aging (Albany NY).

[30] Lipingzuo/chestAge. GitHub.

