# Supplementary material for: Deep Learning–Based Estimated Pulmonary Biological Age From Chest Computed Tomography Images in Healthy Adults: Model Development and Validation Study
Source: JMIR Aging. 2026 Mar 12;9:e78243. doi: 10.2196/78243 (PMC12981372; doi:10.2196/78243)
Supplement: Multimedia Appendix 1 [file aging-v9-e78243-s001.docx]

**Deep Learning-based Estimated Pulmonary Biological Age from Chest CT Images in Healthy Adults: a model development and validation study**

**SUPPLEMENTARY METHODS**

**Model development and test**

A total of 10 deep learning models (Visual Geometry Group (11 layers) [VGG11]+ Long Short-Term Memory (LSTM), Residual Network (18 layers) [Resnet18]+LSTM, ConvNeXt+LSTM, Vision Transformer (ViT)+LSTM, Shifted Windows Transformer (Swin) Transformer+LSTM, 3D VGG11, 3D Resnet18, 3D ConvNeXt, 3D ViT, 3D Swin Transformer), including 4 major categories (2D Convolutional Neural Network (CNN) /Transformer+LSTM, 3D CNN/ Transformer), were trained to predict the age of relatively healthy individuals. Select the best-performing model for further analysis.

The deep learning model connecting a 2D CNN (VGG11[1], ResNet18[2], ConvNeXt[3])+LSTM network was constructed to process sequential image data. We accurately identified and extracted the lung region of interest (ROI) from the whole CT scan using image segmentation. The extracted lung ROIs were spatially resampled to unify their voxel spacing to (1mm, 1mm, 1mm), and the grayscale of interest was adjusted using the lung window (-800, 1600 HU) value to emphasize the features of lung tissues to reduce the interference from other tissue regions. Next, 25 slices were extracted uniformly in the sagittal position direction, and each slice was combined with the upper and lower adjacent slices to form a 3-channel image, similar to the structure of an RGB image. Lastly, adaptive scaling is performed on each image, the long side is scaled to 224pix, and the short side is less than 224pixels for padded black. The 2D CNN network structure to extract the spatial features of each image and obtain 512-dimensional feature vector. For each sequence data, a 25*512 feature vector is obtained, which is used as input to LSTM, which learns the temporal information in the sequence data and finally output ePBA. Sequential chest image features extracted by the convolutional backbone are fed into an LSTM module. The LSTM module consists of two stacked bidirectional layers, each containing 256 hidden units, with 50% dropout applied between layers to mitigate overfitting. The hidden state is reset at the beginning of each sequence to ensure independence across sequences. The LSTM output is mapped to a single prediction via a fully connected layer and normalized using a Sigmoid activation. This design captures both spatial information in individual frames and temporal dependencies across the sequence, providing an effective representation of sequential chest images.

We further incorporated two representative Transformer-based architectures—ViT[4] and Swin Transformer[5]—as alternative feature extractors. The Vision Transformer (ViT) adopts a pure Transformer architecture for vision tasks. Each input image is divided into fixed-size patches of 16×16 pixels, which are then linearly projected into a 384-dimensional embedding space. A learnable class token is prepended to the patch sequence, and positional embeddings are added to retain spatial information. This sequence is processed through 12 Transformer encoder layers, each comprising an 8-head multi-head self-attention (MHSA) mechanism and a feed-forward network (FFN) with a hidden dimension of 1536. Layer normalization and residual connections are employed in each sub-layer to stabilize training and facilitate gradient flow, allowing ViT to effectively capture long-range spatial dependencies across the entire image.The Swin Transformer introduces a hierarchical design to enhance efficiency for high-resolution inputs. The input image is initially partitioned into 4×4 non-overlapping patches, which are embedded and processed in a stage-wise manner. Self-attention is computed within local, non-overlapping windows of size 7×7 to reduce computational complexity. Across four stages, the embedding dimension progressively increases (from 96 to 192, then 384, and finally 768), accompanied by spatial downsampling through patch merging layers that halve the feature map resolution. A shifted window mechanism is applied in alternate layers to enable cross-window connections, thereby facilitating global information propagation while preserving local inductive biases. In our implementation, we utilized the base configurations of ViT-Small (vit_small_patch16_224) and Swin-Tiny (swin_tiny_patch4_window7_224) from the TIMM library (https://github.com/huggingface/pytorch-image-models)to strike a balance between model capacity and computational efficiency. Both Transformer backbones were seamlessly integrated with the LSTM module: features extracted from each frame (via average global pooling followed by a linear projection to a 512-dimensional latent space) form the input sequence to the bidirectional LSTM, enabling end-to-end joint spatial-temporal representation learning.

We evaluated five representative 3D backbones: 3D VGG11[6], 3D ResNet-18[7], 3D ConvNeXt[8], 3D ViT[9], and 3D Swin Transformer [10]. Each backbone extracts spatiotemporal features directly from the 3D volume, followed by global average pooling and a linear head that projects to a single output activated via sigmoid for regression. The VGG11-inspired 3D CNN employs a series of 3D convolutions with progressively increasing channel depths. It initiates with a 3D convolution layer expanding from 3 to 64 channels using a 3×3×3 kernel and padding of 1, followed by max-pooling with a 1×2×2 stride. Subsequent blocks deepen the network: a 3D convolution from 64 to 128 channels, followed by 2×2×2 pooling; two consecutive 3D convolutions from 128 to 256 channels each, followed by pooling; two from 256 to 512 channels, followed by pooling; and two additional layers from 512 to 512 channels, with a final pooling operation incorporating padding on the spatial dimensions. The resulting flattened features, spanning 32,768 dimensions, are fed into three fully connected layers sequentially reducing to 4,096 dimensions, then again to 4,096, and finally to 1 output dimension, with ReLU activations and 50% dropout applied throughout. Weights for the 3D convolutions are initialized using the Kaiming method to promote stable training. The 3D ResNet-18 architecture extends the residual design to volumetric inputs. It utilizes basic residual blocks featuring 3D convolutions with kernels of 1×3×3 or 3×3×3 and incorporates skip connections to alleviate vanishing gradients. The initial stem processes the input to 64 channels, succeeded by four stages with downsampling applied via strides of 2 on the spatial dimensions while preserving depth: each stage comprises two blocks, yielding channel dimensions of 64, 128, 256, and 512. Batch normalization in 3D and ReLU activations follow each convolution, with global average pooling over the full volume preceding the final linear classifier. The 3D ConvNeXt adapts the contemporary ConvNeXt paradigm for volumetric efficiency, prioritizing depthwise-separable convolutions and layer scaling. A stem 3D convolution expands from 3 to 96 channels using a 3×4×4 kernel with strides of 1×4×4 on the spatial dimensions, followed by four stages with depths of three, three, nine, and three blocks, respectively, and channel dimensions of 96, 192, 384, and 768. Each stage consists of ConvNeXt3D blocks, which include a 7×7×7 depthwise 3D convolution, channel-last adapted layer normalization, a GELU-activated pointwise multilayer perceptron with an expansion ratio of 4, and a learnable scale parameter initialized to 1e-6. Inter-stage downsampling employs 1×2×2 3D convolutions, maintaining depth invariance. Global average pooling across depth, height, and width, coupled with layer normalization, precedes the linear head. The 3D ViT harnesses a pure Transformer architecture for processing volumetric patches. The input volume is partitioned into patches of 8×16×16, which are linearly embedded into a 512-dimensional hidden space. Convolutional positional embeddings are incorporated, and the patch sequence is processed through 12 encoder layers, each integrating an 8-head multi-head self-attention mechanism and a feed-forward network with an expansion factor of 4. Layer normalization and residual connections facilitate robust modeling of long-range dependencies across the 3D space. A classification token aggregates the features for the terminal linear projection.The 3D Swin Transformer implements a hierarchical shifted-window attention scheme for efficient 3D processing. Patches are initialized at 2×4×4 with an embedding dimension of 48 and window sizes of 2×7×7. Four stages handle the features with depths of two, two, six, and two blocks, head counts of three, six, twelve, and twenty-four, and progressive dimension growth from 48 to 96, 192, 384, and 768 via patch merging that halves spatial resolution while preserving depth. Relative positional biases and shifted windows enable cross-region interactions without incurring the quadratic complexity of full self-attention. Features from the final stage undergo adaptive 3D average pooling, followed by the linear classifier.

**Reference**

1. Simonyan K, Zisserman A. Very deep convolutional networks for large-scale image recognition[C]// International Conference on Learning Representations (ICLR). 2015.
2. He K, Zhang X, Ren S, Sun J. Deep residual learning for image recognition[C]// Proceedings of the IEEE Conference on Computer Vision and Pattern Recognition (CVPR). 2016: 770–778.
3. Liu Z, Mao H, Wu C Y, Feichtenhofer C, Darrell T, Xie S. A ConvNet for the 2020s[C]// Proceedings of the IEEE/CVF Conference on Computer Vision and Pattern Recognition (CVPR). 2022: 11976–11986.
4. Dosovitskiy A, Beyer L, Kolesnikov A, et al. An image is worth 16x16 words: Transformers for image recognition at scale[J]. arXiv preprint arXiv:2010.11929, 2020.
5. Liu Z, Lin Y, Cao Y, et al. Swin transformer: Hierarchical vision transformer using shifted windows[C]//Proceedings of the IEEE/CVF international conference on computer vision. 2021: 10012-10022.
6. Tran D, Bourdev L, Fergus R, et al. Learning spatiotemporal features with 3d convolutional networks[C]//Proceedings of the IEEE international conference on computer vision. 2015: 4489-4497.
7. Hara K, Kataoka H, Satoh Y. Can spatiotemporal 3d cnns retrace the history of 2d cnns and imagenet?[C]//Proceedings of the IEEE conference on Computer Vision and Pattern Recognition. 2018: 6546-6555.
8. Liu Z, Mao H, Wu C Y, et al. A ConvNeXt for the 2020s[C]//Proceedings of the IEEE/CVF conference on computer vision and pattern recognition. 2022: 11976-11986.
9. Perera S, Navard P, Yilmaz A. Segformer3d: an efficient transformer for 3d medical image segmentation[C]//Proceedings of the IEEE/CVF Conference on Computer Vision and Pattern Recognition. 2024: 4981-4988.
10. Cai Y, Long Y, Han Z, et al. Swin Unet3D: a three-dimensional medical image segmentation network combining vision transformer and convolution[J]. BMC medical informatics and decision making, 2023, 23(1): 33.

**SUPPLEMENTARY TABLES**

**Table S1:** **CT acquisition protocols for different scanners**

| **Device name** | **Matrix** | **Collimation (mm)** | **Pitch** | **Slice thickness (mm)** | **The field of view (mm²)** | **Tube voltage (kV)** | **Tube current (mAs)** |
| --- | --- | --- | --- | --- | --- | --- | --- |
| Siemens Force | 512×512 | 192×0.6 | 1 | 1 | 300×300 | 120 | Auto |
| SOMATOM Definition AS | 512×512 | 128×0.6 | 1 | 1 | 300×300 | 120 | Auto |
| Philips Incisive CT | 512×512 | 60×0.625 | 1 | 1 | 350×350 | 120 | Auto |
| Philips Ingenuity CT | 512×512 | 64×0.6 | 0.797 | 1 | 350×350 | 120 | 163-252 |
| Philips IQon Spectral CT | 512×512 | 64×0.625 | 1.234 | 5 | 400×400 | 120 | 103 |
| United Imaging uCT710 | 512×512 | 40 | 1.0875 | 1 | 350×350 | 120 | Auto |
| GE Revolution CT | 512×512 | 64×0.625 | 0.992 | 5 | 350×350 | 120 | 200-400 |
| GE Discovery CT750 HD | 512×512 | 64×0.6 | 0.758 | 5 | 350×350 | 120 | 250 |

**Table S2** Regression performance parameters of VGG11+LSTM deep learning model

|  | Training dataset | Tuning dataset | Internal test datasets | External test datasets | |
| --- | --- | --- | --- | --- | --- |
|  | Institution A | Institution A | Institution A | Institution B | Institution C |
| **Total individuals** |  |  |  |  |  |
| *r* | 0.99(0.99, 0.99) | 0.97(0.96, 0.97) | 0.97(0.96, 0.97) | 0.97(0.96, 0.97) | 0.98(0.96, 0.99) |
| R^2^ | 0.97(0.97, 0.97) | 0.94(0.93, 0.95) | 0.94(0.93, 0.94) | 0.93(0.92, 0.94) | 0.93(0.84, 0.94) |
| MAE | 2.66(2.58, 2.74) | 3.83(3.55, 4.11) | 3.96(3.75, 4.17) | 4.59(4.34, 4.83) | 3.64(3.47, 3.81) |
| MSE | 11.89(11.78, 12.01) | 23.70(23.26, 24.15) | 25.91(25.59, 26.24) | 34.46(34.12, 34.8) | 21.64(21.37, 21.91) |
| RMSE | 3.45(3.33, 3.56) | 4.87(4.42, 5.31) | 5.09(4.76, 5.42) | 5.87(5.53, 6.21) | 4.65(4.38, 4.92) |
| **Male** |  |  |  |  |  |
| *r* | 0.99(0.99, 0.99) | 0.97(0.96, 0.98) | 0.97(0.97, 0.98) | 0.96(0.95, 0.97) | 0.98(0.96, 0.99) |
| R^2^ | 0.97(0.97, 0.98) | 0.94(0.92, 0.96) | 0.95(0.93, 0.96) | 0.92(0.91, 0.93) | 0.90(0.84, 0.90) |
| MAE | 2.73(2.61, 2.84) | 3.94(3.53, 4.36) | 3.87(3.56, 4.18) | 4.75(4.40, 5.10) | 3.66(3.45, 3.86) |
| MSE | 12.64(12.47, 12.8) | 24.77(24.09, 25.44) | 24.69(24.22, 25.16) | 37.49(37.0, 37.97) | 21.57(21.24, 21.9) |
| RMSE | 3.55(3.39, 3.72) | 4.98(4.30, 5.65) | 4.97(4.50, 5.44) | 6.12(5.64, 6.61) | 4.64(4.31, 4.97) |
| **Female** |  |  |  |  |  |
| *r* | 0.98(0.98, 0.99) | 0.96(0.95, 0.97) | 0.96(0.95, 0.97) | 0.97(0.96, 0.97) | 0.96(0.93, 0.98) |
| R^2^ | 0.97(0.97, 0.97) | 0.93(0.91, 0.95) | 0.92(0.91, 0.94) | 0.94(0.93, 0.95) | 0.94(0.89, 0.98) |
| MAE | 2.60(2.49, 2.70) | 3.73(3.35, 4.10) | 4.04(3.74, 4.33) | 4.40(4.06, 4.73), | 3.61(3.31, 3.91) |
| MSE | 11.20(11.04, 11.36) | 22.79(22.2, 23.37) | 26.94(26.48, 27.4) | 30.89(30.42, 31.37) | 21.78(21.3, 22.25) |
| RMSE | 3.35(3.19, 3.50) | 4.77(4.19, 5.36) | 5.19(4.73, 5.65) | 5.56(5.08, 6.03) | 4.67(4.19, 5.14) |

**Note:** *r* correlation coefficient, R^2^ coefficient of determination, MAE mean absolute error, MSE mean square error, RMSE root mean square error

All the data in parentheses are 99% confidence intervals.

**Table S3** Regression performance parameters of ResNet-18+LSTM deep learning model

|  | Training dataset | Tuning dataset | Internal test datasets | External test datasets | |
| --- | --- | --- | --- | --- | --- |
|  | Institution A | Institution A | Institution A | Institution B | Institution C |
| **Total individuals** |  |  |  |  |  |
| *r* | 1.00(1.00, 1.00) | 0.97(0.96, 0.98) | 0.97(0.97, 0.97) | 0.97(0.96, 0.97) | 0.88(0.87, 0.89) |
| R^2^ | 0.99(0.99, 0.99) | 0.94(0.93, 0.95) | 0.94(0.94, 0.95) | 0.93(0.92, 0.94) | 0.78(0.75, 0.80) |
| MAE | 1.40(1.36, 1.45) | 3.61(3.35, 3.88) | 3.44(3.24, 3.63) | 5.00(4.76, 5.24) | 3.80(3.63, 3.97) |
| MSE | 3.51(3.44, 3.57) | 21.31(20.89, 21.74) | 20.66(20.36, 20.96) | 38.01(37.66, 38.36) | 22.77(22.52, 23.03) |
| RMSE | 1.87(1.81, 1.94) | 4.62(4.19, 5.04) | 4.54(4.25, 4.84) | 6.17(5.82, 6.51) | 4.77(4.52, 5.03) |
| **Male** |  |  |  |  |  |
| *r* | 1.00(1.00, 1.00) | 0.97(0.97, 0.98) | 0.97(0.97, 0.98) | 0.96(0.95, 0.97) | 0.88(0.87, 0.90) |
| R^2^ | 0.99(0.99, 0.99) | 0.95(0.93, 0.96) | 0.95(0.94, 0.96) | 0.92(0.91, 0.93) | 0.78(0.75, 0.81) |
| MAE | 1.43(1.37, 1.49) | 3.55(3.18, 3.92) | 3.50(3.20, 3.80) | 5.12(4.78, 5.46) | 3.83(3.63, 4.04) |
| MSE | 3.47(3.38, 3.56) | 20.41(19.81, 21.0) | 21.87(21.42, 22.32) | 40.1(39.60, 40.59) | 22.81(22.5, 23.12) |
| RMSE | 1.86(1.77, 1.96) | 4.52(3.92, 5.11) | 4.68(4.22, 5.13) | 6.33(5.84, 6.83) | 4.78(4.47, 5.09) |
| **Female** |  |  |  |  |  |
| *r* | 0.99(0.99, 1.00) | 0.97(0.96, 0.97) | 0.97(0.96, 0.97) | 0.97(0.96, 0.98) | 0.87(0.84, 0.89) |
| R^2^ | 0.99(0.99, 0.99) | 0.93(0.91, 0.95) | 0.94(0.93, 0.95) | 0.94(0.93, 0.95) | 0.76(0.71, 0.80) |
| MAE | 1.39(1.32, 1.45) | 3.67(3.29, 4.05) | 3.38(3.12, 3.64) | 4.85(4.51, 5.19) | 3.74(3.44, 4.04) |
| MSE | 3.54(3.45, 3.63) | 22.18(21.58, 22.79) | 19.61(19.21, 20.01) | 35.55(35.07, 36.04) | 22.7(22.25, 23.15) |
| RMSE | 1.88(1.79, 1.97) | 4.71(4.1, 5.32) | 4.43(4.03, 4.83) | 5.96(5.48, 6.45) | 4.76(4.31, 5.21) |

**Note:** *r* correlation coefficient, R^2^ coefficient of determination, MAE mean absolute error, MSE mean square error, RMSE root mean square error

All the data in parentheses are 99% confidence intervals

**Table S4** Regression performance parameters of ConvNeXt+LSTM deep learning model

|  | Training dataset | Tuning dataset | Internal test datasets | External test datasets | |
| --- | --- | --- | --- | --- | --- |
|  | Institution A | Institution A | Institution A | Institution B | Institution C |
| **Total individuals** |  |  |  |  |  |
| *r* | 0.97(0.97, 0.98) | 0.95(0.94, 0.96) | 0.95(0.94, 0.96) | 0.95(0.95, 0.96) | 0.83(0.81, 0.85) |
| R^2^ | 0.95(0.95, 0.95) | 0.90(0.88, 0.92) | 0.91(0.89, 0.92) | 0.91(0.90, 0.92) | 0.69(0.66, 0.72) |
| MAE | 3.57(3.47, 3.66) | 4.64(4.34, 5.02) | 4.80(4.55, 5.05) | 6.78(6.46, 7.10) | 4.86(4.65, 5.08) |
| MSE | 19.49(19.33, 19.64) | 35.35(34.8, 35.9) | 36.81(36.42, 37.21) | 68.96(68.57, 69.36) | 37.48(37.15, 37.80) |
| RMSE | 4.41(4.26, 4.57) | 5.95(5.40, 6.50) | 6.07(5.67, 6.46) | 8.30(7.91, 8.70) | 6.12(5.80, 6.45) |
| **Male** |  |  |  |  |  |
| *r* | 0.98(0.97, 0.98) | 0.95(0.94, 0.97) | 0.95(0.94, 0.96) | 0.95(0.94, 0.96) | 0.83(0.81, 0.85) |
| R^2^ | 0.95(0.95, 0.96) | 0.91(0.89, 0.93) | 0.91(0.89, 0.92) | 0.90(0.88, 0.91) | 0.69(0.65, 0.73) |
| MAE | 3.55(3.41, 3.68) | 4.85(4.35, 5.35) | 5.02(4.65, 5.39) | 6.82(6.38, 7.26) | 5.06(4.79, 5.33) |
| MSE | 19.53(19.31, 19.75) | 36.87(36.04, 37.70) | 39.26(38.65, 39.87) | 70.59(70.03, 71.14) | 40.13(39.73, 40.53) |
| RMSE | 4.42(4.20, 4.64) | 6.07(5.24, 6.90) | 6.27(5.66, 6.88) | 8.40(7.85, 8.95) | 6.33(5.93, 6.73) |
| **Female** |  |  |  |  |  |
| *r* | 0.97(0.97, 0.97) | 0.94(0.93, 0.96) | 0.95(0.94, 0.96) | 0.96(0.95, 0.96) | 0.82(0.79, 0.85) |
| R^2^ | 0.94(0.94, 0.95) | 0.89(0.86, 0.91) | 0.90(0.88, 0.92) | 0.92(0.90, 0.93) | 0.67(0.62, 0.73) |
| MAE | 3.59(3.46, 3.71) | 4.54(4.07, 5.01) | 4.62(4.29, 4.95) | 6.73(6.27, 7.18) | 4.46(4.10, 4.81) |
| MSE | 19.44(19.23, 19.65) | 34.03(33.3, 34.77) | 34.76(34.24, 35.28) | 67.05(66.50, 67.61) | 32.09(31.54, 32.64) |
| RMSE | 4.41(4.20, 4.62) | 5.83(5.10, 6.57) | 5.90(5.38, 6.42) | 8.19(7.63, 8.75) | 5.66(5.12, 6.21) |

**Note:** *r* correlation coefficient, R^2^ coefficient of determination, MAE mean absolute error, MSE mean square error, RMSE root mean square error

All the data in parentheses are 99% confidence intervals

**Table S5** Regression performance parameters of ViT+LSTM deep learning model

|  | Training dataset | Tuning dataset | Internal test datasets | External test datasets | |
| --- | --- | --- | --- | --- | --- |
|  | Institution A | Institution A | Institution A | Institution B | Institution C |
| **Total individuals** |  |  |  |  |  |
| *r* | 0.99(0.99, 0.99) | 0.97(0.96, 0.97) | 0.97(0.96, 0.97) | 0.95(0.95, 0.96) | 0.85(0.84, 0.87) |
| R^2^ | 0.98(0.98, 0.98) | 0.93(0.92, 0.94) | 0.93(0.92, 0.94) | 0.90(0.89, 0.91) | 0.66(0.62, 0.69) |
| MAE | 2.15(2.09, 2.21) | 3.90(3.64, 4.16) | 3.92(3.72, 4.12) | 5.02(4.80, 5.27) | 4.32(4.12, 4.52) |
| MSE | 7.87(7.39, 8.30) | 24.41(21.41, 28.01) | 25.80(22.94, 29.20) | 39.53(36.30, 43.38) | 29.59(26.75, 32.26) |
| RMSE | 2.80(2.71, 2.88) | 4.94(4.63, 5.29) | 5.08(4.79, 5.40) | 6.29(6.03, 6.21) | 5.44(5.17, 5.68) |
| **Male** |  |  |  |  |  |
| *r* | 0.99(0.99, 0.99) | 0.97(0.96, 0.98) | 0.96(0.96, 0.97) | 0.95(0.94, 0.96) | 0.86(0.84, 0.88) |
| R^2^ | 0.98(0.97, 0.98) | 0.94(0.93, 0.95) | 0.93(0.91, 0.94) | 0.89(0.87, 0.90) | 0.67(0.62, 0.72) |
| MAE | 2.09(2.00, 2.18) | 4.02(3.64, 4.40) | 3.85(3.59, 4.16) | 5.02(4.67, 5.37) | 4.20(3.98, 4.44) |
| MSE | 7.51(6.97, 8.15) | 24.94(20.92, 28.93) | 24.72(21.55, 28.73) | 40.55(35.49, 45.93) | 28.44(25.56, 31.90) |
| RMSE | 2.74(2.64, 2.85) | 4.99(4.57, 5.38) | 4.97(4.64, 5.36) | 6.37(5.96, 6.78) | 5.33(5.06, 5.65) |
| **Female** |  |  |  |  |  |
| *r* | 0.99(0.99, 0.99) | 0.96(0.95, 0.97) | 0.97(0.96, 0.97) | 0.96(0.95, 0.96) | 0.84(0.80, 0.87) |
| R^2^ | 0.98(0.98, 0.98) | 0.92(0.90, 0.94) | 0.94(0.92, 0.95) | 0.90(0.89, 0.91) | 0.60(0.50, 0.68) |
| MAE | 2.22(2.13, 2.31) | 3.79(3.44, 4.17) | 4.00(3.70, 4.34) | 5.02(4.66, 5.37), | 4.57(4.25, 4.91) |
| MSE | 8.25(7.50, 8.98) | 23.95(19.34, 29.14) | 27.09(22.42, 33.69) | 38.34(33.56, 43.39) | 31.91(27.53, 36.90) |
| RMSE | 2.87(2.74, 3.00) | 4.89(4.40, 5.40) | 5.20(4.73, 5.80) | 6.19(5.79, 6.59) | 5.65(5.25, 6.07) |

**Note:** *r* correlation coefficient, R^2^ coefficient of determination, MAE mean absolute error, MSE mean square error, RMSE root mean square error

All the data in parentheses are 99% confidence intervals.

**Table S6** Regression performance parameters of Swin Transformer+LSTM deep learning model

|  | Training dataset | Tuning dataset | Internal test datasets | External test datasets | |
| --- | --- | --- | --- | --- | --- |
|  | Institution A | Institution A | Institution A | Institution B | Institution C |
| **Total individuals** |  |  |  |  |  |
| *r* | 0.99(0.99, 1.00) | 0.98(0.98, 0.98) | 0.98(0.97, 0.98) | 0.96(0.96, 0.97) | 0.88(0.86, 0.89) |
| R^2^ | 0.99(0.99, 0.99) | 0.96(0.95, 0.97) | 0.96(0.95, 0.96) | 0.89(0.88, 0.91) | 0.73(0.69, 0.76) |
| MAE | 1.52(1.48, 1.56) | 2.96(2.74, 3.18) | 3.12(2.96, 3.31) | 5.07(4.83, 5.36) | 3.78(3.61, 3.95) |
| MSE | 3.93(3.69, 4.18) | 14.65(12.68, 16.51) | 16.96(14.91, 20.20) | 41.05(37.02, 45.95) | 23.37(21.50, 25.56) |
| RMSE | 1.98(1.92, 2.05) | 3.83(3.56, 4.06) | 4.12(3.86, 4.49) | 6.41(6.08, 6.78) | 4.83(4.64, 5.06) |
| **Male** |  |  |  |  |  |
| *r* | 0.99(0.99, 1.00) | 0.98(0.98, 0.98) | 0.98(0.97, 0.98) | 0.96(0.95, 0.96) | 0.88(0.87, 0.90) |
| R^2^ | 0.99(0.99, 0.99) | 0.96(0.95, 0.97) | 0.95(0.94, 0.96) | 0.88(0.85, 0.90) | 0.72(0.68, 0.76) |
| MAE | 1.47(1.41, 1.53) | 3.20(2.86, 3.51) | 3.13(2.91, 3.38) | 5.22(4.84, 5.56) | 3.85(3.64, 4.03) |
| MSE | 3.64(3.37, 8.92) | 16.50(13.78, 19.20) | 16.28(14.13, 18.59) | 44.26(38.13, 51.08) | 23.89(21.51, 26.50) |
| RMSE | 1.91(1.83, 1.98) | 4.06(3.71, 4.38) | 4.03(3.76, 4.31) | 6.65(6.17, 7.15) | 4.89(4.64, 5.15) |
| **Female** |  |  |  |  |  |
| *r* | 0.99(0.99, 1.00) | 0.98(0.97, 0.98) | 0.98(0.97, 0.98) | 0.97(0.96, 0.97) | 0.86(0.83, 0.89) |
| R^2^ | 0.99(0.99, 0.99) | 0.96(0.94, 0.97) | 0.96(0.94, 0.97) | 0.90(0.89, 0.92) | 0.72(0.65, 0.78) |
| MAE | 1.57(1.51, 1.63) | 2.76(2.48, 3.06) | 3.10(2.83, 3.42) | 4.89(4.53, 5.29), | 3.64(3.33, 3.98) |
| MSE | 4.24(3.87, 4.68) | 13.05(10.53, 16.13) | 17.77(14.04, 23.53) | 37.27(32.58, 42.92) | 22.34(18.63, 26.45) |
| RMSE | 2.06(1.97, 2.16) | 3.61(3.24, 4.02) | 4.22(3.75, 4.85) | 6.10(5.71, 6.55) | 4.73(4.32, 5.14) |

**Note:** *r* correlation coefficient, R^2^ coefficient of determination, MAE mean absolute error, MSE mean square error, RMSE root mean square error

All the data in parentheses are 99% confidence intervals. **Table S7** Regression performance parameters of 3D VGG11 deep learning model

|  | Training dataset | Tuning dataset | Internal test datasets | External test datasets | |
| --- | --- | --- | --- | --- | --- |
|  | Institution A | Institution A | Institution A | Institution B | Institution C |
| **Total individuals** |  |  |  |  |  |
| *r* | 0.99 (0.99, 0.99) | 0.97 (0.96, 0.97) | 0.96 (0.96, 0.97) | 0.95 (0.95, 0.96) | 0.85 (0.83, 0.86) |
| R^2^ | 0.98 (0.98, 0.98) | 0.93 (0.92, 0.94) | 0.92 (0.91, 0.93) | 0.91 (0.89, 0.92) | 0.58 (0.51, 0.63) |
| MAE | 1.94 (1.89, 2.00) | 3.96 (3.69, 4.27) | 4.18 (3.98, 4.40) | 4.58 (4.32, 4.85) | 4.77 (4.56, 4.99) |
| MSE | 6.36 (6.04, 6.72) | 25.19 (21.70, 28.75) | 28.90 (25.94, 32.63) | 35.34 (31.65, 39.98) | 36.37 (33.12, 40.38) |
| RMSE | 2.52 (2.46, 2.59) | 5.02 (4.66, 5.36) | 5.38 (5.09, 5.71) | 5.95 (5.63, 6.32) | 6.03 (5.75, 6.35) |
| **Male** |  |  |  |  |  |
| *r* | 0.99 (0.99, 0.99) | 0.97 (0.96, 0.98) | 0.96 (0.96, 0.97) | 0.94 (0.93, 0.95) | 0.84 (0.82, 0.86) |
| R^2^ | 0.98 (0.98, 0.99) | 0.94 (0.93, 0.95) | 0.93 (0.91, 0.94) | 0.89 (0.86, 0.91) | 0.56 (0.48, 0.62) |
| MAE | 1.97 (1.89, 2.05) | 3.95 (3.55, 4.35) | 4.22 (3.89, 4.52) | 4.92 (4.57, 5.31) | 4,93 (4.69, 5.21) |
| MSE | 6.57 (6.04, 7.12) | 24.31 (19.53, 29.19) | 30.49 (25.85, 36.04) | 41.45 (35.22, 48.75) | 38.57 (35.08, 42.44) |
| RMSE | 2.56 (2.46, 2.67) | 4.93 (4.42, 5.40) | 5.52 (5.08, 6.00) | 6.44 (5.93, 6.98) | 6.21 (5.92, 6.51) |
| **Female** |  |  |  |  |  |
| *r* | 0.99 (0.99, 0.99) | 0.96 (0.95, 0.97) | 0.96 (0.95, 0.97) | 0.96 (0.96,0.97) | 0.86 (0.82, 0.89) |
| R^2^ | 0.98 (0.98, 0.98) | 0.91 (0.89, 0.93) | 0.92 (0.90, 0.93) | 0.93 (0.91, 0.94) | 0.60 (0.48, 0.70) |
| MAE | 1.92 (1.84, 2.00) | 3.97 (3.56, 4.38) | 4.16 (3.91, 4.43) | 4.18 (3.87, 4.53) | 4.45 (4.10, 4.80) |
| MSE | 6.17 (5.74, 6.68) | 25.95 (20.88, 31.27) | 27.57 (24.47, 31.23) | 28.16 (23.96, 32.63) | 31.90 (26.47, 38.90) |
| RMSE | 2.48 (2.40, 2.58) | 5.09 (4.57, 5.59) | 5.25 (4.95, 5.59) | 5.31 (4.90, 5.71) | 5.65 (5.15, 6.24) |

**Note:** *r* correlation coefficient, R^2^ coefficient of determination, MAE mean absolute error, MSE mean square error, RMSE root mean square error

All the data in parentheses are 99% confidence intervals.

**Table S8** Regression performance parameters of 3D Resnet18 deep learning model

|  | Training dataset | Tuning dataset | Internal test datasets | External test datasets | |
| --- | --- | --- | --- | --- | --- |
|  | Institution A | Institution A | Institution A | Institution B | Institution C |
| **Total individuals** |  |  |  |  |  |
| *r* | 1.00 (1.00, 1.00) | 0.96 (0.95, 0.97) | 0.96 (0.95, 0.96) | 0.95 (0.94, 0.96) | 0.84 (0.83, 0.86) |
| R^2^ | 0.99 (0.99, 0.99) | 0.92 (0.91, 0.93) | 0.92 (0.91, 0.93) | 0.90 (0.89, 0.91) | 0.61 (0.55, 0.66) |
| MAE | 1.41 (1.35, 1.47) | 4.23 (3.95, 4.58) | 4.33 (4.10, 4.57) | 4.90 (4.65, 5.17) | 4.56 (4.35, 4.77) |
| MSE | 3.39 (3.14, 3.62) | 29.04 (25.32, 33.91) | 31.06 (27.62, 35.01) | 38.54 (34.95, 42.74) | 33.81 (30.74, 37.25) |
| RMSE | 1.84 (1.77, 1.90) | 5.39 (5.03, 5.82) | 5.57 (5.25, 5.92) | 6.21 (5.91, 6.54) | 5.82 (5.54,6.10) |
| **Male** |  |  |  |  |  |
| *r* | 1.00 (1.00, 1.00) | 0.96 (0.96, 0.96) | 0.95 (0.95, 0.96) | 0.94 (0.93, 0.95) | 0.84 (0.82,0.86) |
| R^2^ | 0.99 (0.99, 0.99) | 0.93 (0.91, 0.94) | 0.91 (0.89, 0.92) | 0.88 (0.86, 0.89) | 0.59 (0.52, 0.65) |
| MAE | 1.41 (1.35, 1.47) | 4.36 (3.88, 4.80) | 4.27 (3.99, 4.55) | 5.30 (4.95, 5.65) | 4.68 (4.41, 4.94) |
| MSE | 3.39 (3.14, 3.62) | 31.94 (25.25, 38.61) | 29.97 (25.82, 34.25) | 44.69 (38,90, 50.74) | 35.42 (31.69, 39.42) |
| RMSE | 1.84 (1.77, 1.90) | 5.65 (5.03, 6.21) | 5.47 5.08, 5.85) | 6.69 (6.24, 7.12) | 5.95 (5.63, 6.28) |
| **Female** |  |  |  |  |  |
| *r* | 1.00 (1.00, 1.00) | 0.96 (0.95, 0.96) | 0.96 (0.95, 0.97) | 0.96 (0.95, 0.97) | 0.84 (0.80, 0.87) |
| R^2^ | 0.99 (0.99, 0.99) | 0.91 (0.89, 0.93) | 0.92 (0.91, 0.94) | 0.92 (0.91, 0.93) | 0.62 (0.52, 0.70) |
| MAE | 1.47 (1.42, 1.54) | 4.11 (3.88, 4.80) | 4.41 (4.07, 4.76) | 4.42 (4.13, 4.78) | 4.30 (3.98, 4.66) |
| MSE | 3.58 (3.32, 3.86) | 26.51 (21.89, 31.61) | 32.37 (26.60, 39.68) | 31.31 (27.57, 35.56) | 30.55 (25.77, 36.08) |
| RMSE | 1.89 (1.82, 1.96) | 5.15 (4.68, 5.62) | 5.69 (5.16, 6.30) | 5.60 (5.25, 5.96) | 5.53 (5.08, 6.01) |

**Note:** *r* correlation coefficient, R^2^ coefficient of determination, MAE mean absolute error, MSE mean square error, RMSE root mean square error

All the data in parentheses are 99% confidence intervals.

**Table S9** Regression performance parameters of 3D ConvNeXt deep learning model

|  | Training dataset | Tuning dataset | Internal test datasets | External test datasets | |
| --- | --- | --- | --- | --- | --- |
|  | Institution A | Institution A | Institution A | Institution B | Institution C |
| **Total individuals** |  |  |  |  |  |
| *r* | 0.96 (0.96, 0.97) | 0.91 (0.90, 0.93) | 0.89 (0.88, 0.90) | 0.89 (0.88, 0.91) | 0.71 (0.67, 0.74) |
| R^2^ | 0.93 (0.93, 0.93) | 0.83 (0.81, 0.86) | 0.79 (0.76, 0.82) | 0.80 (0.77, 0.82) | 0.10 (-0.04, 0.22) |
| MAE | 3.97 (3.85, 4.08) | 6.23 (5.82, 6.69) | 6.80 (6.43, 7.17) | 6.80 (6.46, 7.19) | 6.78 (6.47, 7.12) |
| MSE | 26.12 (24.63, 27.64) | 61.23 (53.83, 69.43) | 77.73 (69.11, 86.82) | 78.01 (68.96, 87.46) | 77.56 (70.11, 85.93) |
| RMSE | 5.11 (4.96, 5.26) | 7.82 (7.34, 8.33) | 8.82 (8.31, 9.32) | 8.83 (8.30, 9.35) | 8.81 (8.37, 9.27) |
| **Male** |  |  |  |  |  |
| *r* | 0.97 (0.96, 0.97) | 0.91 (0.89, 0.93) | 0.90 (0.88, 0.91) | 0.87 (0.84, 0.90) | 0.71 (0.66, 0.74) |
| R^2^ | 0.93 (0.93, 0.94) | 0.83 (0.80, 0.87) | 0.80 (0.77, 0.83) | 0.76 (0.72, 0.80) | 0.06 (-0.14, 7.32) |
| MAE | 4.02 (3.88, 4.18) | 6.71 (5.94, 7.42) | 7.09 (6.59, 7.61) | 7.21 (6.68,7.73) | 6.91 (6.53, 7.32) |
| MSE | 26.87 (24.97, 28.94) | 71.42 (57.26, 86.42) | 82.46 (70.39, 95.32) | 88.04 (74.62, 101.72) | 81.61 (71.90, 93.04) |
| RMSE | 5.18 (5.00, 5.38) | 8.45 (7.57, 9.30) | 9.08 (8.39, 9.76) | 9.38 (8.64, 10.09) | 9.03 (8.48, 9.65) |
| **Female** |  |  |  |  |  |
| *r* | 0.96 (0.96, 0.96) | 0.91 (0.89, 0.93) | 0.89 (0.86, 0.91) | 0.91 (0.89, 0.93) | 0.69 (0.62, 0.74) |
| R^2^ | 0.92 (0.92, 0.93) | 0.82 (0.78, 0.86) | 0.78 (0.73, 0.82) | 0.83 (0.79, 0.86) | 0.13 (-0.11, 0.33) |
| MAE | 3.92 (3.75, 4.07) | 5.82 (5.29, 6.34) | 6.56 (6.06, 7.07) | 6.31 (5.84, 6.79) | 6.49 (57.34, 82.33) |
| MSE | 25.42 (23.43, 27.68) | 52.37 (44.07, 61.26) | 73.77 (61.29, 88.00) | 66.21 (55.99, 77.68) | 69.33 (57.34, 82.33) |
| RMSE | 5.04 (4.84, 5.26) | 7.24 (6.64, 7.83) | 8.59 (7.83, 9.38) | 8.14 (7.48, 8.81) | 8.33 (7.57, 9.07) |

**Note:** *r* correlation coefficient, R^2^ coefficient of determination, MAE mean absolute error, MSE mean square error, RMSE root mean square error

All the data in parentheses are 99% confidence intervals.

**Table S10** Regression performance parameters of 3D ViT deep learning model

|  | Training dataset | Tuning dataset | Internal test datasets | External test datasets | |
| --- | --- | --- | --- | --- | --- |
|  | Institution A | Institution A | Institution A | Institution B | Institution C |
| **Total individuals** |  |  |  |  |  |
| *r* | 0.95 (0.95, 0.96) | 0.87 (0.85, 0.89) | 0.88 (0.86, 0.89) | 0.86 (0.85, 0.88) | 0.64 (0.60, 0.67) |
| R^2^ | 0.91 (0.90, 0.91) | 0.76 (0.72, 0.80) | 0.77 (0.74, 0.79) | 0.74 (0.71, 0.77) | -0.01 (-0.14, 0.12) |
| MAE | 4.64 (4.52, 4.76) | 7.26 (6.72, 7.76) | 7.45 (7.10, 7.84) | 8.04 (7.62, 8.44) | 7.33 (7,02, 7.67) |
| MSE | 33.63 (31.91, 35.42) | 88.11 (74.76, 101.70) | 88.22 (80.03, 97.00) | 99.82 (90.17, 110.19) | 86.75 (79.62, 94.39) |
| RMSE | 5.80 (5.64, 5.95) | 9.39 (8.65, 10.08) | 9.39 (8.95, 9.85) | 9.99 (9.50, 10.50) | 9.31 (8.92, 9.72) |
| **Male** |  |  |  |  |  |
| *r* | 0.95 (0.95, 0.95) | 0.87 (0.83, 0.90) | 0.86 (0.84, 0.88) | 0.85 (0.82, 0.87) | 0.63 (0.59, 0.66) |
| R^2^ | 0.90 (0.89, 0.91) | 0.76 (0.69, 0.81) | 0.74 (0.70, 0.78) | 0.71 (0.67, 0.75) | -0.07 (-0.23, 0.07) |
| MAE | 4.53 (4.37, 4.68) | 7.81 (6.93, 8.71) | 7.35 (6.85, 7.84) | 8.26 (7.69, 8.81) | 7.66 (7.28, 8.09) |
| MSE | 31.76 (29.73, 33.85) | 103.16 (79.89, 128.11) | 86.02 (74.79, 98.40) | 104.80 (91.84, 119.00) | 92.82 (83.95, 102.44) |
| RMSE | 5.64 (5.45, 5.82) | 10.16 (8.94, 11.32) | 9.27 (8.65, 9.92) | 10.24 (9.58, 10.91) | 9.63 (9.16, 10.12) |
| **Female** |  |  |  |  |  |
| *r* | 0.96 (0.95, 0.96) | 0.87 (0.84, 0.89) | 0.89 (0.87, 0.91) | 0.88 (0.85, 0.90) | 0.63 (0.56, 0.69) |
| R^2^ | 0.91 (0.91, 0.92) | 0.75 (0.69, 0.80) | 0.78 (0.75, 0.82) | 0.76 (0.72, 0.79) | 0.07 (-0.20, 0.27) |
| MAE | 4.77 (4.61, 4.95) | 6.78 (6.07, 7.48) | 7.58 (6.99, 8.11) | 7.77 (7.22, 8.35) | 6.67 (6.17, 7.24) |
| MSE | 35.64 (33.01, 38.23) | 75.01 (60.13, 91.82) | 90.85 (77.98, 102.60) | 93.97 (82.30, 110.81) | 74.43 (63.29, 89.24) |
| RMSE | 5.97 (5.75, 6.18) | 8.66 (7.75, 9.58) | 9.53 (8.83, 10.13) | 9.69 (9.07, 10.53) | 8.63 (7.96, 9.45) |

**Note:** *r* correlation coefficient, R^2^ coefficient of determination, MAE mean absolute error, MSE mean square error, RMSE root mean square error

All the data in parentheses are 99% confidence intervals.

**Table S11** Regression performance parameters of 3D Swin Transformer deep learning model

|  | Training dataset | Tuning dataset | Internal test datasets | External test datasets | |
| --- | --- | --- | --- | --- | --- |
|  | Institution A | Institution A | Institution A | Institution B | Institution C |
| **Total individuals** |  |  |  |  |  |
| *r* | 1.00 (1.00, 1.00) | 0.94 (0.93, 0.95) | 0.93 (0.92, 0.94) | 0.92 (0.91, 0.93) | 0.78 (0.75, 0.80) |
| R^2^ | 0.99 (0.99, 0.99) | 0.88 (0.86, 0.90) | 0.87 (0.85, 0.88) | 0.85 (0.83, 0.87) | 0.34 (0.23, 0.42) |
| MAE | 1.45 (1.41, 1.50) | 5.16 (4.79, 5.55) | 5.49 (5.22, 5.80) | 5.87 (5.55, 6.20) | 5.90 (5.63, 6.17) |
| MSE | 3.57 (3.39, 3.77) | 44.23 (37.89, 50.78) | 48.91 (44.16, 54.66) | 57.80 (51.21, 64.28) | 57.21 (52.05, 63.42) |
| RMSE | 1.89 (1.84, 1.94) | 6.65 (6.15, 7.13) | 6.99 (6.65, 7.39) | 7.60 (7.16, 8.02) | 7.56 (7.21, 7.96) |
| **Male** |  |  |  |  |  |
| *r* | 0.99 (0.99, 1.00) | 0.94 (0.92, 0.95) | 0.93 (0.92, 0.94) | 0.90 (0.89, 0.92) | 0.77 (0.75, 0.80) |
| R^2^ | 0.99 (0.99, 0.99) | 0.88 (0.85, 0.91) | 0.86 (0.84, 0.88) | 0.82 (0.78, 0.85) | 0.30 (0.18, 0.41) |
| MAE | 1.40 (1.35, 1.46) | 5.53 (4.87, 6.17) | 5.36 (5.00, 5.73) | 6.36 (5.92, 6.80) | 6.14 (5.76, 6.50) |
| MSE | 3.38 (3.13, 3.65) | 51.66 (40.59, 63.27) | 45.23 (39.85, 51.28) | 67.02 (57.41, 76.86) | 60.61 (54.42, 67.35) |
| RMSE | 1.84 (1.77, 1.91) | 7.19 (6.37, 7.95) | 6.73 (6.31, 7.16) | 8.189(7.58, 8.77) | 7.79 (7.38, 8.21) |
| **Female** |  |  |  |  |  |
| *r* | 1.00 (1.00, 1.00) | 0.94 (0.92, 0.95) | 0.94 (0.92, 0.95) | 0.94 (0.92, 0.95) | 0.78 (0.72, 0.83) |
| R^2^ | 0.99 (0.99, 0.99) | 0.87 (0.84, 0.90) | 0.87 (0.85, 0.89) | 0.88 (0.85, 0.90) | 0.37 (0.15, 0.53) |
| MAE | 1.51 (1.44, 1.57) | 4.83 (4.38, 5.34) | 5.65 (5.21, 6.11) | 5.30 (4.87, 5.72) | 5.42 (4.95, 5.92) |
| MSE | 3.77 (3.51,4.07) | 37.77 (31.19, 45.50) | 53.30 (45.40, 62.03) | 46.96 (39.17, 55.99) | 50.30 (40.70, 65.66) |
| RMSE | 1.94 (1.87, 2.02) | 6.15 (5.59, 6.75) | 7.30 (6.74, 7.88) | 6.85 (6.25, 7.48) | 7.09 (6.38, 8.10) |

**Note:** *r* correlation coefficient, R^2^ coefficient of determination, MAE mean absolute error, MSE mean square error, RMSE root mean square error

All the data in parentheses are 99% confidence intervals.

**Table S12** Variance inflation factor (VIF) analysis.

| **Variables** | **Tolerance** | **VIF** | **Variables** | **Tolerance** | **VIF** |
| --- | --- | --- | --- | --- | --- |
| Age gap | 0.62 | 1.61 | Age gap | 0.62 | 1.63 |
| Chronological age | 0.60 | 1.66 | Chronological age | 0.57 | 1.76 |
| Sex | 0.94 | 1.06 | Sex | 0.90 | 1.11 |
| Body mass index | 0.92 | 1.08 | Body mass index | 0.84 | 1.20 |
| mMRC | 0.92 | 1.08 | mMRC | 0.92 | 1.09 |
|  |  |  | Smoking status | 0.96 | 1.05 |
|  |  |  | Hypertension | 0.92 | 1.09 |
|  |  |  | Diabetes | 0.89 | 1.12 |
|  |  |  | Cerebrovascular disease | 0.86 | 1.16 |

**Note:** mMRC, the modified Medical Research Council scale*.*

**Table S13** Schoenfeld residual test results for the Cox proportional hazards models.

| **Model 1** | **χ²** | ***P* value** | **Model 2** | **χ²** | ***P* value** | **Model 3** | **χ²** | ***P* value** |
| --- | --- | --- | --- | --- | --- | --- | --- | --- |
| Age gap | 0.75 | .39 | Age gap | 0.13 | .72 | Age gap | 0.02 | .89 |
|  |  |  | Chronological age | 0.70 | .40 | Chronological age | 0.27 | .61 |
|  |  |  | Sex | 1.06 | .30 | Sex | 0.87 | .35 |
|  |  |  | Body mass index | 0.75 | .39 | Body mass index | 0.80 | .37 |
|  |  |  | mMRC | 0.25 | .62 | mMRC | 0.38 | .54 |
|  |  |  |  |  |  | Smoking status | 3.95 | .14 |
|  |  |  |  |  |  | Hypertension | 1.84 | .17 |
|  |  |  |  |  |  | Diabetes | 0.06 | .81 |
|  |  |  |  |  |  | Cerebrovascular disease | 0.41 | .52 |
| **GLOBAL** | 0.75 | .39 | **GLOBAL** | 2.65 | .75 | **GLOBAL** | 10.27 | .42 |

**Note:** mMRC, the modified Medical Research Council scale*.*

**SUPPLEMENTARY FIGURES**

**
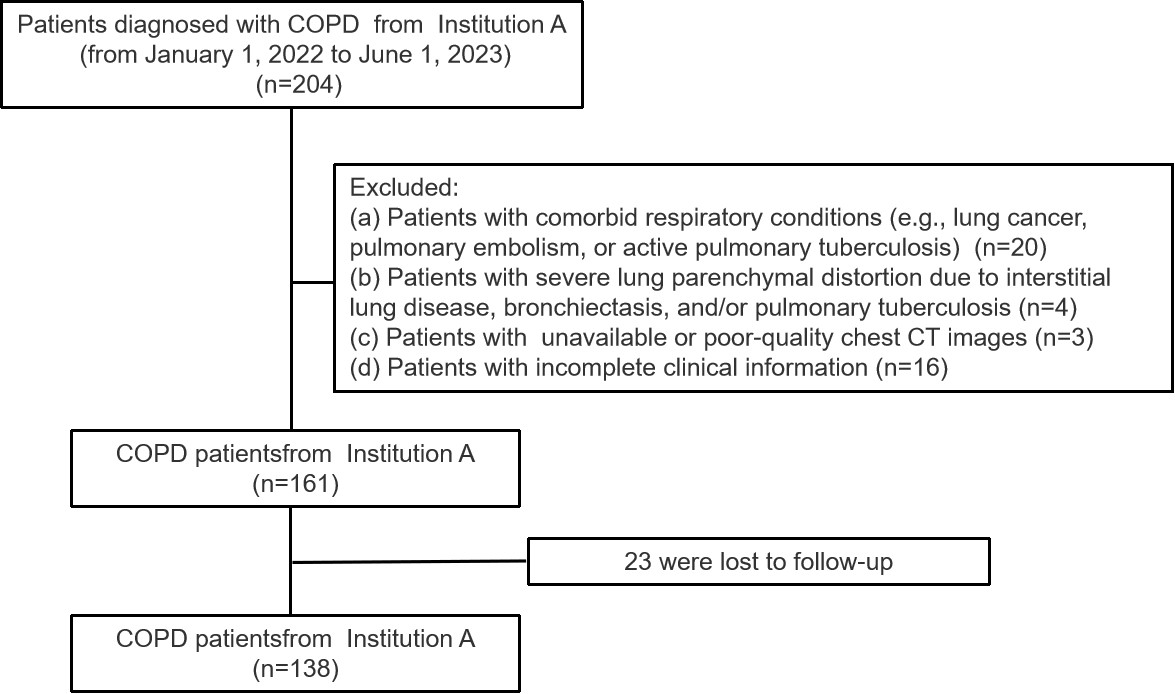
**

**Figure S1.** The flowchart of included and excluded COPD patients in model application.
